# Couples Coping Together: A Scoping Review of the Quantitative and Qualitative Evidence and Conceptual Work Across Three Decades

**DOI:** 10.3389/fpsyg.2022.876455

**Published:** 2022-06-10

**Authors:** Katharina Weitkamp, Guy Bodenmann

**Affiliations:** Clinical Psychology for Children/Adolescents and Couples/Families, University of Zurich, Zurich, Switzerland

**Keywords:** dyadic coping, couple coping, close relationships, scoping review, stress

## Abstract

Dyadic coping (DC), how couples cope together to deal with a stressor like chronic illness, has received increased attention over the last three decades. The aim of the current study was to summarize the current state of research on DC in couples. We conducted a scoping review of qualitative, quantitative, and mixed-methods studies published between 1990 and 2020, assessing DC in couples during three decades. 5,705 studies were identified in three electronic databases and hand searches. We included 643 sources in this review (with a total of *N* = 112,923 study participants). Most studies were based in the global North, particularly in the US and Europe. Publication numbers increased constantly over time. A third of study designs were cross-sectional studies followed by qualitative and longitudinal studies. The most prolific DC research areas were related to DC and minor stressors and DC and major physical health stressors. Overall, DC has been established internationally as a highly relevant construct in many disciplines (clinical, social, developmental, personality psychology, social work, nursing etc.). To conclude, the review reveals that future studies should focus on predictors, trajectories, and the importance of very specific DC behaviors for personal and dyadic functioning.

## Statement of Relevance

This review is of great importance because, for the first time, it not only provides an overview of studies in specific areas (e.g., cancer research, health impairments, and well-being) or meta-analyses (e.g., on DC and relationship satisfaction), but attempts to provide an overall view of the research field. Thus, it provides a unique opportunity to gain an informed view of the evolution of DC approaches, their dissemination and development, as well as their application in the field.

## Introduction

### Historical Evolution of the Dyadic Coping Concept

Thirty years ago, stress and coping were viewed as purely individual phenomena, albeit originating from exchanges with the social environment (Lazarus and Folkman, 1984). Most scholars of that time did not consider coping with stress as either a dyadic or a social process. Only in the 1990ies, scholars in Europe and the United States began to question this individual-oriented understanding of stress and coping. They pointed out that these phenomena in close, committed relationships affect both partners due to their interdependence. Dealing with stress is therefore not an isolated but shared experience, concerning the well-being, satisfaction, and general functioning of both partners involved (Bodenmann, 1997). Since the early 1990ies, a vast expansion of research in the area has occurred. While in 1992, four publications referred to dyadic coping, in 2020, more than 5,000 publications were published on dyadic coping or couples’ coping according to Web of Knowledge. Dyadic coping (DC) research has turned into a prolific interdisciplinary research field including disciplines such as clinical, developmental, personality, health, social psychology, social work, health sciences, family science, nursing, and medicine.

Leading theoretical contributions have guided the field, originating in the early 1990ies by the *Coping congruence* (Revenson, 1994); the *Relationship-focused Model* (RFM; DeLongis and O’Brien, 1990; Coyne and Smith, 1991), or the *Systemic-Transactional Model* (STM; Bodenmann, 1995, 2005). Later, the *communal coping model* (CCM; Lyons et al., 1998), the *Developmental-contextual Model* (DCM; Berg and Upchurch, 2007), and the *Relational-Cultural Coping Model* (RCCM; Kayser et al., 2007) followed. For an integrative overview of the different DC models see Falconier and Kuhn (2019) and for theoretical frameworks on couples’ coping with chronic illness see Revenson and DeLongis (2011).

All approaches are united by their view of shared or mutual stress experiences (shared stress appraisals by both partners; Bodenmann, 1995, 1997; Lyons et al., 1998). Instead of seeing the individuals’ sole responsibility for their stress (their problem), DC acknowledges that a stressor is affecting the couple as a whole and is appraised as a shared task demanding both partners’ involvement and shared actions. This understanding of stress opens the option for DC, where either one partner supports the other in their own coping efforts (supportive or delegated DC) or both partners get involved in shared problem-solving or joint emotion-regulation (i.e., common DC, communal DC, and collaborative DC) (Bodenmann, 1997; Lyons et al., 1998; Berg and Upchurch, 2007). Among the different DC approaches, the STM received most international recognition and is the most widespread theoretical model guiding research. Therefore, most studies that are reported in this review are based on this approach. In order to facilitate reading, we do, however, not distinguish between the different DC approaches but refer to them as a unit (as they all share the notion of interdependence; Falconier and Kuhn, 2019).

While the DC-literature developed independently from the social support literature, both approaches started at a similar time addressing support processes among couples (e.g., Cutrona and Suhr, 1992). Nevertheless, until recently, both lines of research developed side by side without seeking integration, albeit an effort of theoretical incorporation of similarities and differences would be rewarding (Cutrona et al., 2018). While in the 1990ies a multitude of differences distinguished both approaches, today it is above all the notion of common dyadic coping which differentiates most between DC approaches and social support literature. For theoretical clarity, in this review we focus exclusively on DC-studies as only DC-approaches address common, joint or collaborative DC that proved to be especially important above and beyond traditional support.

Two main research lines can be detected in DC-studies, already since the emergence of this research field. On the one hand, the focus on minor stressors like daily hassles (Lazarus and Folkman, 1984; i.e., stressors related to work load, unpleasant neighbors, stressful everyday experiences, worries with children, subjects that were predominately studied in Europe), on the other hand, the focus on major life events or health issues that characterized early DC-research in the United States. Major stressors are normative or non-normative critical life events, such as severe illness, handicap, unemployment, death of a significant other, or a serious accident (Dohrenwend and Dohrenwend, 1974). Mainly, studies on severe physical illness (Weitkamp et al., 2021) or disability (Bertschi et al., 2021) represent this line of research, with a clear predominance of cancer studies (e.g., Regan et al., 2015; Traa et al., 2015; Badr and Acitelli, 2017; Ştefǎnuţ et al., 2021). In both continents, the role of DC on relationship satisfaction, couple’s communication or interaction, sexuality, commitment, or general well-being were assessed in the majority of these studies.

Apart from the mere prediction of individual or relational outcomes by DC, this research also emphasizes dynamic and processual features of stress and coping by studying stress spill-over and cross-over processes within couples and how couples effectively cope with these stressors (Story and Bradbury, 2004; Bodenmann et al., 2006, 2007; Neff and Karney, 2007; Randall and Bodenmann, 2009; Falconier et al., 2015a; Nguyen et al., 2020). In this context, also the role of DC as mediator (e.g., Donato et al., 2014) or moderator (e.g., Falconier et al., 2013; Merz et al., 2014) was explored.

Within this line of inquiry, more recently, stress coping processes were subjected to microanalytical scrutiny (e.g., Kuhn et al., 2017, 2018; Pagani et al., 2019). More recently, researchers also started to explore DC in different cultures (Falconier et al., 2016; Hilpert et al., 2016), age groups (Landis et al., 2013; Acquati and Kayser, 2019) and couples dealing with minority stress (Meuwly et al., 2013; Mitchell et al., 2015; Randall et al., 2017a,b; Song et al., 2020; Meuwly and Davila, 2021; Sarno et al., 2021).

### Key Assumptions of the Dyadic Coping Concept and Forms

Dyadic coping is an additional and complementary concept to individual coping or social support (Bodenmann et al., 2016; Falconier and Kuhn, 2019). According to Bodenmann et al. (2016) dyadic coping processes involve:

– *cognitive* components: individual and dyadic appraisals of stress and coping resources, individual and dyadic goals within the coping process.
– *emotional* components: shared emotions, emotional contagion, and co-regulation of emotions.
– *physiological* components: shared arousal and interdependent physiological reactions, impact of DC on endocrine processes.
– *behavioral* aspects: e.g., overt stress management activities, active listening to the partner’s stress-related self-disclosure, verbal and non-verbal support behaviors like holding each other, hugging, giving a massage, and active joint problem-solving.

In the Systemic Transactional Model (STM; Bodenmann, 1997, 2005), DC is considered as a process in which three factors of stress communication and coping operate and interact: (a) the stress expression of one partner (verbally, non-verbally, and paraverbally), (b) the perception and interpretation of these signals by the other partner, and (c) the reaction of the other partner to these stress signals. The reaction can either be non-responsive (intentional ignoring the partner’s stress), getting similarly affected (stress contagion) or engaging in positive or negative DC. Dyadic coping includes positive partner-oriented behaviors (e.g., emotion-focused supportive DC or problem-focused supportive DC, delegated DC, and active engagement), forms of negative DC (e.g., hostile, ambivalent or superficial DC, protective buffering or overprotection, controlling) or couple-oriented behaviors (e.g., termed joint/common, collaborative, and communal DC), according to individual and dyadic appraisals, goals, and skills (Bodenmann, 2005; Falconier and Kuhn, 2019).

Dyadic coping may be understood along a continuum of partner involvement from lack of involvement (in the case of health issues, for instance, the patient perceives that they are coping individually and cannot count on their partner), joint problem-solving and shared emotion regulation (Revenson and Hagedoorn, 2019) to over-involvement of the partner (e.g., patient perceives the spouse as controlling, engaging in miscarried helping, overprotecting) (Berg and Upchurch, 2007).

Dyadic coping has *two main functions*, a stress-related and a relationship-related function (Bodenmann, 2005). The stress-related function pertains to the reduction of stress that either affects primarily one partner and spills over to the other partner or that affects both partners at the same time (we-stress). The aim is the maintenance or restoration of the general well-being of both partners. According to the cascade model in STM (Bodenmann, 2005), DC becomes relevant if individual coping was not successful in reducing one’s negative emotions or in solving the problem. The second and maybe more important function of DC is the enhancement of feelings of “we-ness” among partners, their mutual trust and intimacy, their mutual attachment and commitment (Cutrona, 1996; Bodenmann, 2005).

### Assessment of the Dyadic Coping Concept

Dyadic coping is usually assessed by self-reports and validated questionnaires: Perceptions of Collaboration Questionnaire (PCQ; Berg et al., 2008), Dyadic Coping Inventory (DCI; Bodenmann, 2008b), Empathic Responding Scale (ERS; O’Brien and DeLongis, 1996), Relationship Focused Coping Scale (RFCS; Coyne and Smith, 1991), or the use of we-talk (Slatcher and Pennebaker, 2006; Rohrbaugh et al., 2008; Tausczik and Pennebaker, 2010). Several studies are based on daily diaries (e.g., Kuhn et al., 2018; Leuchtmann et al., 2018; Lau et al., 2019; Xu et al., 2019) or coding of overt dyadic coping behavior (Dyadic Coping Coding System; Bodenmann, 2008a; Protective Buffering Observation Coding System; Langer et al., 2007). In most studies, the DCI is used, which allows comparison of findings across different labs and cultures.

### Aims of the Study

The field of couple coping research has clearly caught on and flourished since its inception in the 1990ies. The present scoping review aimed to synthesize research on the interpersonal coping theories and concepts mentioned above to give a broad overview across the last three decades (1990–2020). We included qualitative, quantitative, and mixed-methods studies comprehensively, to provide as complete a picture as possible. Additionally, we aimed to give an overview of the areas of stressors, where DC has been investigated. Although there are some reviews or meta-analyses of DC such an overview has so far not been carried out.

Leading questions of this scoping review are: Based on the current state of DC research, where does the field come from and where is it heading? What areas of research have been covered so far and which seem necessary or fruitful to be tackled in the coming decade?

The research questions were the following: What is the current state of knowledge on DC? Namely, (1) where has research using the DC-approach been conducted (geographical locations)? (2) How is the evolvement of DC-publications over time? (3) In which areas of stressors has DC mainly been investigated? and (4) what kinds of study designs were used to investigate DC?

## Methods

A scoping review of the DC literature was undertaken to capture the complete body of knowledge since the notion of DC first emerged in the early 1990s (among others Bodenmann and Perrez, 1991; Coyne and Smith, 1991; Revenson, 1994; Lyons et al., 1995) until 2020 (covering three decades). We chose the scoping review as a means of data synthesis, since it offers the possibility to integrate a large body of research from different methodologies (quantitative, qualitative, and mixed methods). We conducted the scoping review based on published guidance by Peters et al. (2015). We followed these guidelines in terms of the development of a scoping review protocol defining the objectives and methods, followed by the systematic literature search (detailed below). For the data extraction, which is in scoping reviews referred to as “charting of results,” we developed a charting table which was tested on a number of different study types (qualitative, quantitative, mixed-methods, intervention, and conceptual) to ensure all relevant results and information are extracted. The results are presented as recommended by Peters et al. (2015) showing the distribution of studies by year of publication, country of origin, and research area. Additionally, we created a flow diagram in accordance with the Preffered Reporting Items for Systematic Reviews and Meta-Analyses (PRISMA) methods guidelines (Liberati et al., 2009).

### Literature Search

For the scoping review, we conducted a literature search on March 24, 2021 of the following electronic databases in the field of psychology: *Psycinfo, Psyndex*, and *Medline.* Search terms regarding DC were “dyadic coping,” “communal coping,” “couple coping,” “collaborative coping,” or “relationship-focused coping” in title, abstract, or key words. The search terms were combined with the Boolean operator OR. Beforehand, we conducted a pilot literature search on the theoretical concepts, scanned the key words of these publications. Based on the extraction of these key words relevant to our theoretical focus, we formulated the included search terms.

We used the following inclusion criteria for selecting studies: (a) a focus on DC in romantic couples (or related terms like collaborative coping or relationship-focused coping), (b) publications were released between 1990 and 2020, (c) published in English, German, Spanish, French, or Portuguese because these are the languages with most research output in the field of DC, (f) published as a journal article, book, book chapter, or dissertation. When studies were published in parallel in a dissertation and a journal article, we drew on the journal articles. We deliberately included gray literature such as non-peer reviewed scientific journals, book chapters, and dissertations to allow for the broad spectrum of DC research to be characterized. Overall, we included only studies where the full-text was available with the exception of dissertations. We included 21 dissertations based on their abstract only (if necessary information for data extraction were available from the abstract alone) when the full-text was not available, with the intend to illustrate that a rich body of knowledge exists from dissertations but is lost due to existing publication practices of dissertation theses.

We excluded studies that either dealt only with individual processes or outcomes or reported on social support, spousal support, or dyadic adjustment only. Including the literature of the associated concept of spousal support was beyond the scope of this review (see section “Introduction,” as DC and social support literature still represent two different fields of research). This ensured that only studies were considered that focus on stress expression, dyadic appraisals, or dyadic responsiveness, and the different forms of DC in dealing with minor and major stressors to warrant at least a certain level of homogeneity.

### Study Selection

**FIGURE 1:**
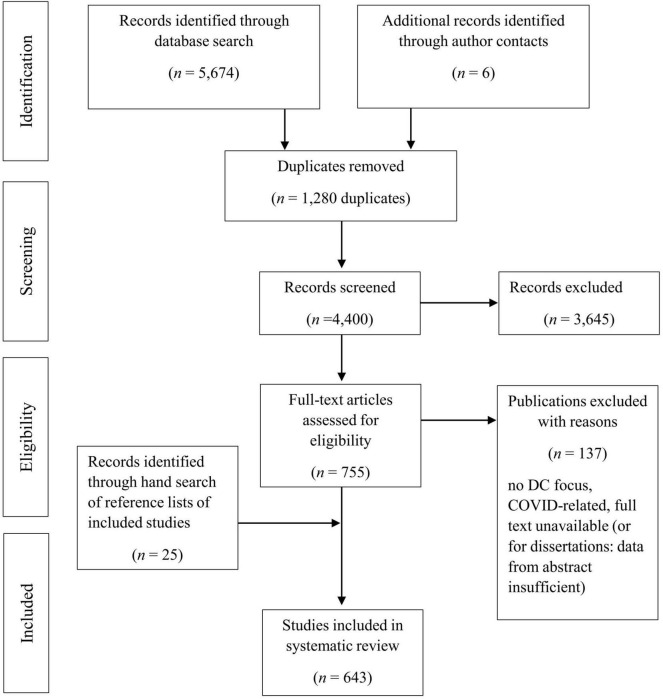
Flow chart.

The initial search yielded 5,674 likely relevant publications. Figure 1 shows the flow chart of the study identification, retrieval, and the number of eligible publications. Codings were carried out by a senior researcher and two PhD students, assisted by graduate students. Data extraction were carried out by one trained person and then checked by another trained researcher, experienced in systematic reviews. If double-checks were showing disagreement, coders consulted with other members of the coding team. Studies were included if an agreement was reached according to the inclusion criteria. We removed 1,280 duplicates and screened the titles and abstracts of the remaining references for eligibility. At this step, 3,645 publications were excluded. In case of insufficient information, references were carried to the next step where full texts were retrieved and screened. When full texts were not available, authors were contacted if we could find a contact address. Unfortunately, quite a number of unavailable sources were dissertations. Six further references could be included thanks to authors who provided us with their full-text publications. After assessing the full texts, 136 additional sources were excluded. Additionally, we excluded one early COVID-related paper, to consistently represent DC pre-pandemic research. Leaving the integration of COVID-related DC research for a later review. At this point, we included 618 publications. Within these publications we hand searched the reference lists and included further 25 references. Thus, the final sample consisted of 643 publications, which form the basis for this review.

### Charting the Results

We entered each publication into a table identifying the authors, title, study design, sample (for longitudinal studies, we drew on sample size at baseline), country of origin, and DC measures. Two researchers extracted data from each paper independently, resolving disagreements by discussion.

Due to the sheer number of included publications, a detailed narrative synthesis was deemed inappropriate. Thus, attention was drawn to basic numerical analysis of the extent, nature and distribution of the studies included in the review. We produced tables and charts and presented the results as different “maps” of the data that align to the objectives and scope of the review (Peters et al., 2015). We collated overviews of the field in terms of distribution of studies by year of publication, geographical area, publication type, interventions, and types of minor/major stressors assessed in relation to DC. For the identification of minor/major stressors, we formed overarching categories of research topics related to DC based on the theoretical differentiation of minor and major stressors (Dohrenwend and Dohrenwend, 1974; Caspi et al., 1987; see also Randall and Bodenmann, 2009). Based on the content of studies, we further organized publications in terms of the research focus: While some reported on stress more general (which we clustered as “undifferentiated”) other publications focused on specific stressors. These specific stressors clustered around three major groups of stressors: (1) physical health conditions, (2) mental health conditions, and (3) child- and parenting-related stress. These categories were then integrated into the framework of minor/major stressors and will be presented in this manner.

## Results

Overall, 643 publications were included. These publications reported data of 137,401 subjects. Since in quite a number of cases, a single study sample lead to multiple publications, elimination of this salient double counting lead to a total sample of *N* = 112,923 individuals. Sample units were mostly couples (*k* = 374^1^; 78.7% of samples), some studies recruited individuals reporting on their relationship (*k* = 78; 16.6% of samples), in some cases a mixed sample of couples and singles participated (*k* = 23; 4.8% of samples). We counted each participant here for the total sample size. Sample sizes ranged from case studies with a single dyad (*n* = 2) to *n* = 7,973 in a large cross-cultural study with data from 35 countries (Hilpert et al., 2016). Sample sizes varied by methodology: Qualitative sample sizes ranged from *n* = 4 to 192 with an average of M = 34.76 (SD = 34.23). Quantitative sample sizes ranged from *n* = 20 to 7,973 with an average of M = 373.33 (SD = 609.79). Sample sizes of mixed-methods designs fell in between with an average of M = 65.29 (Range *n* = 17–145, SD = 45.61). As can be seen from the mean sample sizes, on average sample sizes seemed to be adequate for the specific study design. Even though the minimum sample sizes also suggest that some studies seemed to have been underpowered (for instance, *n* = 20 in quantitative analyses).

### Geographical Location

**FIGURE 2:**
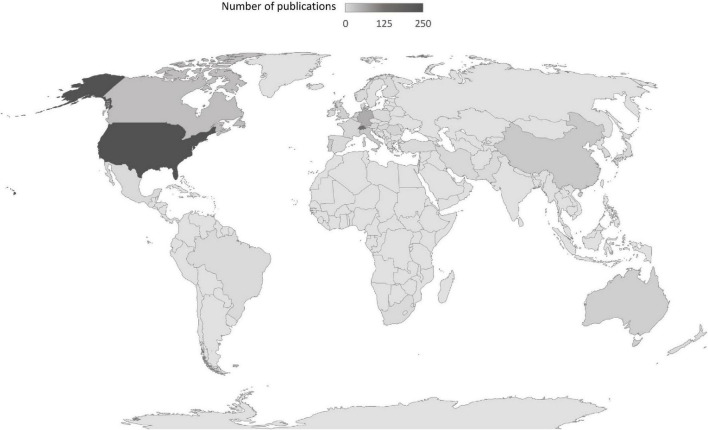
Number of publications about dyadic coping (DC) around the world. Darker areas stand for greater number of publications; light gray areas represent countries without any DC publications (Figure powered by Bing© GeoNames, Microsoft, TomTom, Wikipedia).

Today, DC research is conducted by researchers and labs all over the globe and in five continents (Africa, Asia, Australia, Europe, and Northern America), albeit with a focus in Western Europe (in Switzerland in particular) and the United States. More recently, DC research began to emerge in Asia, namely in China, Iran, South Korea, Japan, and Pakistan (see Figure 2).

The United States were the country with the most publications (245 publications, 38.1%) followed by Switzerland with 144 publications (22.4%) and Germany with 55 publications (8.6%).^2^ A number of Western countries and China had between 34 (5.3%) and 13 publications (2.0%), in descending order: Canada, Italy, China, Portugal, the Netherlands, Australia, and the United Kingdom. No stand-alone publications could be included from most parts of South and Central America, Africa, Russia and former Soviet countries, India, and Southeastern Asia. However, a notable cross-cultural study by Hilpert et al. (2016) included samples from 35 countries from across the globe. In this transcultural study, data on DC and its association with relationship satisfaction were collected for the first time from the following countries: Bulgaria, Croatia, Estonia, Ghana, Indonesia, Kazakhstan, Kenia, Malaysia, Nigeria, Russia, Slovakia, and Saudi Arabia. The authors of this cross-cultural study draw the conclusion that the association between DC and relationship satisfaction was significant in all countries, but varied in strength of the association and also between men and women between different countries, pointing to the relevance of cultural circumstances in which couples’ DC is situated (Hilpert et al., 2016).

### Publications Across Time

**FIGURE 3:**
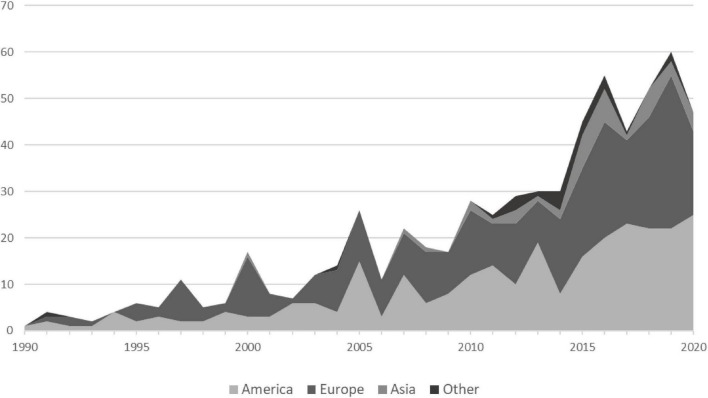
Number of dyadic coping (DC) publications between 1990 and 2020 grouped by continents. The category “Other” includes studies from Africa and Oceania; “America” includes studies from North and South America.

As can be seen in Figure 3, DC research emerged around 1990 in Europe and Northern America almost in parallel with one to 11 publications per year across the first decade. The second decade from 2000 to 2010 saw a gradual increase in publications, again mainly from (Western) Europe and the United States. Annually, seven to 26 publications were published with peaks in 2000, 2005, and 2007. Across the most recent decade, 2010–2020, publications displayed quite a steep increase, showing how the field is currently growing. Between 29 to 60 publications emerged each year. Again, publications were mostly from the United States and Europe, but researchers in Asia as well as Africa and Oceania (presented together as “other” in Figure 3) started to integrate the concept of DC in their research on stress as well with increasing publication numbers since about 2010.

### Publication Type

The majority of publications were journal articles (*k* = 479, 74.5%) and to a lesser degree book chapters (*k* = 80, 12.4%), dissertations (*k* = 72, 11.2%), and books (*k* = 12, 1.9%). We deliberately aimed to include non-peer review journal articles alongside the common practice of peer-reviewed articles. In this way, we intended to be as inclusive of the research field as possible. It is noteworthy, however, that of the 479 article publications (97.9% of journal articles), only 10 were either non-peer reviewed or we could not verify peer-review status (2.0% of journal articles). Thus, for DC publications peer-reviewed publications seem to be standard practice. The most frequent publication outlets were the Journal of Family Psychology (24 publications), Frontiers in Psychology (18 publications), the Journal of Social and Personal Relationships (17 publications), and Psycho-Oncology (14 publications). Overall, DC research was published in 208 different journals, which mirrors the range of subjects that were studied in relation to DC.

It is worth mentioning that of the 72 dissertations only 18 dissertations lead to one or more peer-reviewed journal article publications. Thirty-three dissertations were stand-alone publications. A further 21 doctoral theses were included based only on the abstracts; since we were not able to attain a full-text (three of those were published as journal articles that were included as well). Hence, a rich body of knowledge on DC is not readily accessible.

### Types of Stressors

**FIGURE 4:**
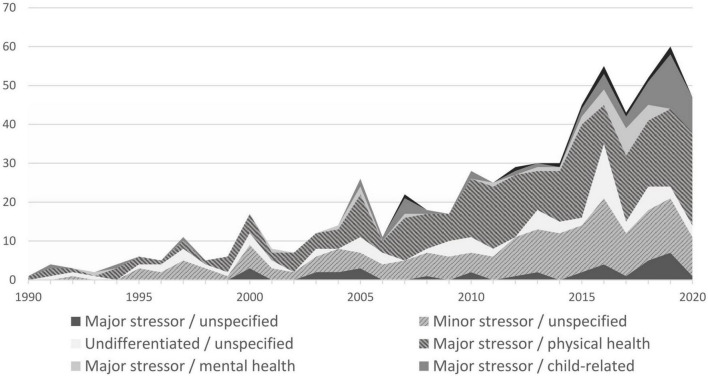
Number of dyadic coping (DC) publications between 1990 and 2020 grouped by types of stressors.

In terms of types of stressors, early publications focused primarily on DC related to major life stressors (see Figure 4). To date, this remains the largest group of scientific publications. Studies on minor stressors emerged more frequently after 1994. However, this focus remained a smaller group compared with research on major stressors. More than half of the publications focused on major stressors (*k* = 374, 58.2%) followed by minor stressors (*k* = 192, 29.9%). A minority of studies either did not differentiate between the types of stressors (*k* = 44, 6.8%) or included both major and minor stressors (*k* = 33, 5.1%). We subdivided stressors further into areas of stressful life incidences that became apparent while charting the results. These areas are physical health, mental health, child-related, and unspecified.

The most prolific field of research were studies related to major physical health stressors with a particularly prolific subgroup of DC research in couples facing a cancer diagnosis. Right from the beginning of DC research, chronic health issues were a central subject of DC research with a gradual and steady increase across the decades. DC and mental health, however, were the focus of publications mainly since 2000 with few studies each year. Additionally, child-related stress and DC were researched right from the 1990s with occasional publications over the decades and a recent increase since 2015 (see Figure 4). Research on minor stressors and DC emerged quite from the beginning (mainly in Europe) with a gradual increase over time. Quite a number of studies did not focus on any particular stressor, but studied major and/or minor stressors in general, which we termed “undifferentiated/unspecified” in Figure 4. This encompasses a small group of publications across time.

### Study Designs

**FIGURE 5:**
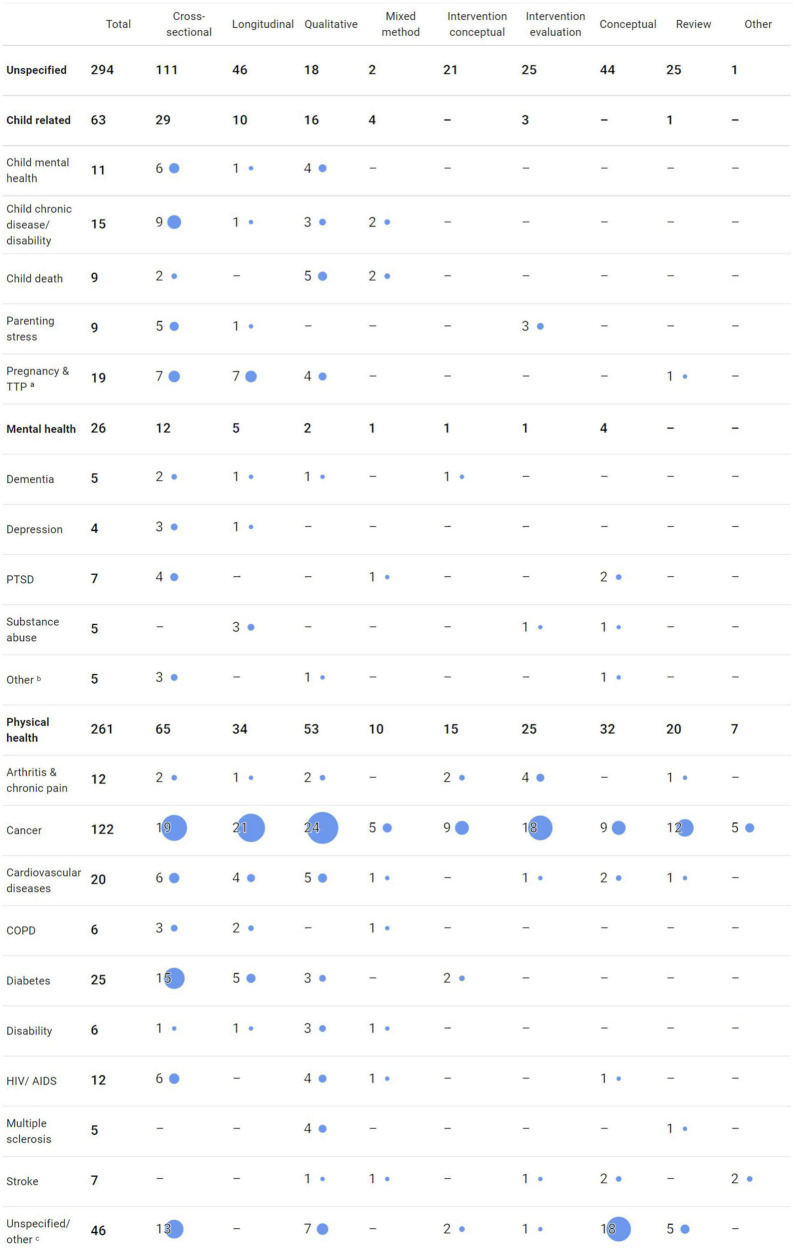
Frequencies of dyadic coping (DC) publications organized by study design and stressor type between 1990 and 2020. *^a^*TTP, transition to parenthood. *^b^*The category “Mental health – Other” includes studies on eating disorders, general mental health condition, self-injury, and sexual functioning. *^c^*The category “Physical health – Unspecified/other” includes studies on cystic fibrosis, endometriosis, fibromyalgia, inflammatory bowel disease, kidney failure, lupus erythematosus, Parkinson’s disease, sleep disorder, and mixed or unspecified health conditions.

In terms of study design, not surprisingly, the largest group with over a quarter of all publications were cross-sectional questionnaire studies (*k* = 187, 29.1%). The next most frequent study designs with over 10% each were qualitative studies (*k* = 89, 13.8%), longitudinal studies (*k* = 85, 13.2%), and conceptual papers (*k* = 80, 12.4%) followed by intervention trials related to efficacy, effectiveness, or feasibility (*k* = 54, 8.4%). Longitudinal studies were mostly follow-up studies (*k* = 73) and less often daily diary studies (*k* = 12). Less current, with just over 5%, were reviews (*k* = 44, 6.8%) and conceptual papers on interventions (*k* = 37, 5.8%). Less frequent with under five percent of all publications were psychometric validation of DC measures (mainly international validations of the Dyadic Coping Inventory, DCI, Bodenmann, 2008b), mixed-methods studies, experiments, case studies, and study protocols. Interestingly, only two meta-analyses seem to exist to date. See Figure 5 for details.

### Types of Stressors by Study Design

To attain a comprehensive overview of the field of DC research, we plotted types of stressors against study design (see Figure 5). This overview is informative in depicting which areas received broad scientific attention with varied methodological approaches and which areas are still in need of research attention.

The largest group of DC publications were on understanding DC in general, couples dealing with unspecified minor stressors, or certain mixed topics for instance work stress, financial strain, minority stress or retirement transition. Mostly, those publications were either cross-sectional, longitudinal, or conceptual in nature (*k* = 110, 47, 44, respectively). Quite a number of DC interventions for minor stressors have been published in either conceptual (*k* = 21) or evaluative publications (*k* = 25). With 25 reviews, facets of general DC in couples have been mapped out to some extend already.

For child-related stressors, studies were mostly cross-sectional (*k* = 29) or qualitative (k = 16) and focused mainly on DC in pregnancy and the transition to parenthood (TTP, *k* = 19) or DC with child health or disability issues (*k* = 15). Interventions were evaluated only in relation to parenting stress (*k* = 3). Only one review was published on DC and pregnancy and TTP.

In the realm of couples facing mental health issues, the empirical evidence is scattered across study designs and mental disorders. Mostly, studies focused on posttraumatic stress disorders (PTSD, *k* = 7), substance abuse (*k* = 5), dementia (*k* = 5), or depression (*k* = 4). To date, no review exists in this area. Only two publications dealt with DC interventions for couples with one partner suffering from a mental health condition (one conceptual, one intervention evaluation).

Chronic physical health conditions were the largest group of studies (*k* = 261). Here, overall, an aggregation of studies in the field of cancer (*k* = 122) was visible with quite a large number of qualitative, longitudinal, and cross-sectional publications. Additionally, by far the largest group of conceptual and evaluative papers on DC interventions were located in cancer research in addition to cross-sectional, longitudinal, and qualitative research. Furthermore, diabetes (*k* = 25) and cardiovascular diseases (*k* = 20) received sizable research attention. Another prolific area were conceptual (*k* = 13) and cross-sectional studies (*k* = 18) with unspecified health conditions. Most published reviews addressed either cancer or chronic physical health conditions in general.

### Dyadic Coping Assessment

Most studies relied on self-report questionnaires to assess DC. Widely used was the Dyadic Coping Inventory (DCI; Bodenmann, 2008b). This questionnaire is available in 24 languages and validated in 15 languages. Other measurement instruments were the Relationship-Focused Coping Scale (RFCS; Coyne and Smith, 1991) or the Ways of Giving Support (Buunk et al., 1996). A large group of studies used specifically developed idiosyncratic measures for the specific research purposes.

A second strand of operationalization consisted of video/audio recordings of actual DC interaction sequences. Couples were invited to talk about a stressful experience, thus stress expression, listening, and DC could be observed. Analyses mostly drew upon the use of “we”-language, the first-person plural pronoun use (Lyons et al., 1998), behavioral coding of communal coping (Helgeson et al., 2018), DC-processes (Kuhn et al., 2017, 2018).

A new advancement that may be pinpointed here, is the use of contextualized measures, like the dyadic coping inventory for financial stress (Falconier et al., 2019) or goal-based DC (Martos et al., 2019).

### Interventions

Our literature search lead us to include 54 publications on intervention studies including randomized controlled trials (RCTs) as well as pilot studies. Another 37 publications reported conceptual developments of interventions. We observed considerable variability in terms of intervention content, scope, study design, and methodological rigor. To sum it up, variety is the unifying denominator of these studies. The interventions varied in terms of focus on DC. Some interventions were specifically designed to target dyadic coping communication as the main target component of change; others merely included aspects of DC as part of a larger intervention. Interventions targeted two main groups of stressors: minor stressors (*k* = 23 publications) and cancer diagnoses (*k* = 18 publications). Other stressors were chronic physical conditions (chronic pain management, stroke, etc., *k* = 7) and health behavior promotion (weight loss, smoking cessation, etc.) as well as parenting (*k* = 3) and mental health issues (*k* = 1).

Half of the studies were RCTs (*k* = 28), a further nine studies were non-randomized controlled trials and nine were single group pre-post designs without a control condition. Additionally, main outcomes varied from dyadic coping to relationship satisfaction or physical markers for physical conditions. Not surprisingly, in view of the large variability in intervention content and duration, effect sizes showed great range from small to large effects (effect sizes were reported in *k* = 36 publications). Most effects seemed to be in the small to moderate range. Five reviews exist on DC interventions, for instance on woman’s post-partum depression (Alves et al., 2018), various chronic illnesses (Martire et al., 2010), and cancer (Baik and Adams, 2011; Li and Loke, 2014; Luo et al., 2020). The reviews suggest, that couple-oriented interventions have varying effect sizes. Martire et al. (2010) pointed out that effectiveness may be strengthened by targeting partners’ influence on patient health behaviors and focusing on couples with high illness-related conflict, low partner support, or low overall marital quality. Methodological limitations made it difficult to determine whether the inclusion of the partner is associated with intervention efficacy, in particular for postpartum depression studies (Alves et al., 2018).

## Discussion

This scoping review aimed to investigate the current state of research related to dyadic coping in committed relationships. We included 643 publications with almost 113,000 subjects in the review. The field of DC research is fertile and proliferating with an increasing number of publications per year across the last three decades. Interestingly, the most dominant publishing themes were on the understanding and relevance of DC in general, followed by chronic physical health conditions, namely, cancer research.

Regarding study design, the most frequent method were cross-sectional studies. Prospective studies were far less prevalent. It is important to keep in mind that DC is not a static concept, but changes according to the nature of the stressor (see assumption of transaction, STM; Bodenmann, 2005), the couples adjustment (Helgeson, 1993) as well as in situational dynamics (Kuhn et al., 2017). Many factors not only influence DC but are also affected by it in turn. For instance, relationship satisfaction can increase the likelihood of DC (Bodenmann, 1997) and can be further enhanced by DC processes in return (Falconier et al., 2015b). In terms of qualitative studies, quite often these studies did not mention DC explicitly or refrained from referencing well-known DC theoretical frameworks. However, we included these qualitative studies based on the content of the results and the conceptual proximity to the construct. When couples talked about their experiences with minor and major stressors, they often addressed facets of DC, which may serve as a proof of concept, validating DC theories through subjective narratives. Mixed methods research, on the other hand, where the integration of qualitative and quantitative data was the explicit aim of a study, was still scarce. The field of DC research would benefit from more mixed methods studies, insofar as these studies could draw on DC concepts explicitly in the quantitative arm and enrich these with subjective accounts of DC experiences to provide more nuance to DC in different settings and with varying stressors.

Historical and current priorities in the health sector are mirrored in the field of DC research: DC and physical chronic conditions have been studied from the beginning with a continually growing body of evidence particularly for couples affected by cancer. Even though depression is a leading cause of disability worldwide and is a major contributor to the overall global burden of disease (Wang et al., 2007) just like, for instance, cancer (Ferlay et al., 2020), the relevance of mental health conditions have been neglected to date in the health care sector and similarly in DC research. Here it received only marginal attention (Bodenmann et al., 2004, 2008). An increase in publications on DC and mental health stressors was only visible in the last five years and is a welcome and required development. A similar recent increase was noticeable for child-related stressors. Interestingly, mental health issues and child-related stressors may be considered as typical stressors highly affecting the couple relationship, since the challenges and difficulties have a strong interpersonal impact. In our understanding, these stressors are not conceptually different from health or financial stress, according to the transactional stress theory (Lazarus and Folkman, 1984) and its continuation in the STM (Bodenmann, 2005), where the stressor *per se* is less important than the way it is appraised (primary and secondary appraisal). Thus, a further increase in DC research activities in these areas are not only desirable but also necessary and they do not require adaptations of the DC-models.

As noted above, DC research in couples affected by mental health issues is only recently developing. Whether stigmatization of mental health in the public health sector and the society at large were at the root of this research neglect, cannot be answered by this review. However, within the DC and mental health publications that exist so far, another layer of stigmatization is visible when looking at the current blind spots of DC. To date, there were no studies on psychoses or personality disorders. This may reflect the fact that mental disorders are still broadly considered as individual concerns [see also Diagnostic and Statistical Manual (DSM; American Psychiatric Association, 2013); or the International Classification of Diseases (ICD-11; World Health Organization, 2019)] and an interpersonal view is still lacking (Leuchtmann and Bodenmann, 2017).

In terms of chronic physical diseases, disproportionately, the bulk of studies investigated DC in the field of cancer and diabetes. However, this does not mirror the prevalence rates of chronic diseases, which in the Western world are diseases of the nervous system, hypertension, headache/migraine, chronic respiratory disease, genitourinary diseases, osteoathrosis, back and spinal cord disorders, skin diseases, and allergy (Dalstra et al., 2005). Interestingly, no studies were found on common chronic conditions, like headache/migraine, genitourinary diseases, back and spinal cord disorders, skin diseases, or allergy. Even though these conditions may vary in severity, with some chronic illnesses like allergies needing less DC, for other chronic physical illnesses, the lack of couple research is deplorable. For instance, DC regarding headache/migraine, skin diseases or back and spinal cord disorders that are very frequent and burdensome for couples, affecting daily functioning as well as the couples’ sexual life, could contribute to a better understanding of the impact of these demands on couple functioning, but also on couples’ possibilities of coping with them.

In terms of measurement instruments, validated and widely used measures exist (e.g., DCI; Bodenmann, 2008b) alongside a collection of self-developed measures and items (e.g., in the context of diary studies). In future studies, a stricter focus on the utilization of validated scales and a multi-method approach may be beneficial. Additionally, it may be worthwhile to move beyond the reliance on self-reports, as they are not able to capture the dynamic and processual nature of DC. DC research would benefit from more experimental or observational studies that emphasize dynamic and processual aspects. As the STM postulates a process of stress signals, detection of these signals and responses to them, we would need more studies answering questions on how different couples (depending on commitment, relationship satisfaction, age, duration of the relationship, racial and cultural background, sexual orientation, etc.) navigate through these stages and where important switching points in the DC-process are deficient. We have already gained a great deal of knowledge on the importance of DC for general couple functioning (i.e., quality, stability) and well-being of both partners, but the knowledge is sparse regarding dynamic processes, both short-term and long-term, answering questions which factors particularly contribute to successful DC in the interplay of a specific situation, a given context, both partners (i.e., mood, motivation, and skills) and the couple (i.e., relationship satisfaction, commitment, joint goals, etc.).

Thus, there is a lack of studies that illustrate what functional DC looks like in concrete interactions and how DC changes over time (with increasing length of the relationship, within different phases of the relationship or developmental tasks). We also note a gap regarding predictors of DC. DC was rarely included as a dependent variable in the studies, mostly as an independent variable, mediator or moderator. Thus, knowledge on factors that trigger, hinder or enhance DC are only known from theoretical models (Bodenmann, 1995; Bodenmann et al., 2016).

Due to the considerable variability of intervention quality, methodological rigor of effectiveness trials, and utilized outcome measures, reliable conclusions on the efficacy of DC interventions cannot be drawn at this stage. Even though the majority of studies yielded promising small to moderate effect sizes for various outcome measures, a moderate decrease in DC was observed as well in a study on internet-based guided self-help for vaginal penetration difficulties (Zarski et al., 2017).

Most researchers publishing on DC were based in the United States or other nations fitting into the Western, Educated, Industrialized, Rich, and Democratic (WEIRD) nations (Henrich et al., 2010). Even though these countries make up only about 12% of the world’s population, most research has been conducted in these countries, limiting the representativeness and generalization of findings. Comparative research, however, suggests that there is substantial variability in populations and that WEIRD subjects are even particularly unusual compared with the rest of the world’s population (Henrich et al., 2010). This pattern of culturally homogenous samples is common across psychological research. Nevertheless, the intercultural study on DC by Hilpert et al. (2016) in 35 nations or Asian studies (e.g., Xu et al., 2019) suggest that mechanisms of DC are similar in different cultures.

In future research, further efforts should be made to study DC in couples from a wide range of cultural, societal, and ethnic backgrounds (for a new cross-cultural study, see for example Randall et al., 2022). So far, we also know little about binational couples and how they deal together with stress.

Additionally, it is about time to move beyond a heteronormative lens. Some studies focused on gay and lesbian couples, though these identities were mostly assessed in terms of risk and victimization, like HIV prevention or minority stress, or general relationship satisfaction. Those are important issues to address, nonetheless, DC researchers could learn from queer intersectional analyses and be more attentive to inclusivity, intersectionality, and diversity. Although some studies have already been conducted with same-sex couples, there is a real need for increased research activity in these groups. We think that more intersectional research (integrating socioeconomic status, sexual orientation, age groups, with or without children, and type of stressors) might bring new and pioneering insights.

Dyadic coping has been established as a highly relevant construct in many disciplines (clinical, social, developmental, personality psychology, social work, nursing, etc.), as reflected in the increasing number of publications. This is likely to be the case because the construct reaches beyond dyadic communication and individual coping and considers a new, important dimension in close relationships, namely interdependence and how it can be used for thriving close relationships. In the future, it will not only be a matter of anchoring DC in further fields of application, but also of gaining a better understanding of the conditions within couple relationships that enable adequate and flourishing DC in order to make this important resource available. We need to know more about predictors, trajectories, and the importance of very specific DC behaviors for personal and dyadic functioning. This outlines directions and challenges for future research.

### Limitations

A number of limitations pertaining to our review need to be taken into account when considering our findings in addition to the above discussed limitations of the included studies. Firstly, despite great efforts, we could not get hold of several dissertations because either we were unable to contact authors (mainly, because they left the university context and could not be located) or authors failed to respond. Thus, in some areas the body of knowledge may be incomplete in this review. Unfortunately, our futile efforts mirror the difficulty to access the potentially rich body of knowledge that was researched as part of doctoral theses. However, it appeared that providing a full-text version of the dissertation online seems to become more and more common practice at least at major universities. Thus, more doctoral theses were available from recent than from earlier years. Nonetheless, implications for training and mentorship of early-career investigators may be gained. In this sense, cumulative doctoral theses may reach a larger audience and thus create more scientific impact. Alternatively, doctoral positions could be extended beyond the formal doctoral defense to enable early-career investigators to publish their theses in peer-reviewed journals.

We included studies up to the end of December 2020, thus creating a symbolic time frame of three decades, to map out the development of DC research across this period. Incidentally, this cut-off drew a line between pre-COVID and pandemic-related papers. Only one included study published COVID-related data (Williamson, 2020) which we excluded for consistency. Whilst scholars would no doubt argue about our chosen time frame and the exclusion of the COVID-related publication, reviewing pandemic related papers will be left for later reviews.

In some cases, it was difficult to draw a clear line between the inclusion and exclusion of studies from the adjoining or overarching field of spousal support research. Including these as well, was beyond the scope of this review. However, both research areas are intertwined and could enrich each other. More efforts should be made in the future to bring both fields together. Additionally, in future research, increasing efforts should be made to collect longitudinal data, given the dynamic, transactional, and circular nature of DC. Finally, we focused only on DC in intimate relationships. Even though other dyads are relevant for DC as well and gain increasingly attention, like fictive kin, parent and adult child or trainer and elite athlete dyads. In spite of these limitations, a strength of our study was the inclusion also of gray literature (e.g., unpublished dissertations), which is often excluded in systematic reviews, even though they may provide thorough insights.

## Conclusion

Dyadic coping research represents a dynamic field of research that continues to undergo prosperous development. While research on DC was originally conducted mainly in the US and Europe, research activity has expanded to all inhabited continents, especially to Asia in more recent times. In the future, both will be important, to develop DC further theoretically and to highlight the processual structures and the meaning of specific DC behaviors (e.g., which DC behaviors outshine others and are particularly memorable?). To achieve these goals, more mixed methods studies and experimental studies are needed. Furthermore, it would also be important to further embed DC in health promotion and clinical practice and make the potential of this construct available to couples of all backgrounds, ethnicities, ages, and gender orientations.

## Data Availability Statement

The original contributions presented in this study are included in the Supplementary Material, further inquiries can be directed to the corresponding author.

## Author Contributions

KW and GB contributed to the conception and design of the review. KW carried out and supervised the literature search and data extraction, and wrote the first draft of the manuscript. GB wrote sections of the manuscript. Both authors contributed to manuscript revision, read, and approved the submitted version.

## Conflict of Interest

The authors declare that the research was conducted in the absence of any commercial or financial relationships that could be construed as a potential conflict of interest.

## Publisher’s Note

All claims expressed in this article are solely those of the authors and do not necessarily represent those of their affiliated organizations, or those of the publisher, the editors and the reviewers. Any product that may be evaluated in this article, or claim that may be made by its manufacturer, is not guaranteed or endorsed by the publisher.

## Footnotes

1 The percentages relate to the number of studies with samples (*n* = 475 of all publications). We excluded conceptual and review publications for this step of the analysis.

2 In cases where data was collected in more than one country, we refer to the country of the affiliation of the respective first author here.

## References

[B1] Acquati C. Kayser K. (2019). Dyadic coping across the lifespan: a comparison between younger and middle-aged couples with breast cancer. *Front. Psychol.* 10:404. 10.3389/fpsyg.2019.00404 30941068 PMC6433932

[B2] Afifi T. D. Hutchinson S. Krouse S. (2006). Toward a theoretical model of communal coping in postdivorce families and other naturally occurring groups. *Commun. Theor.* 16 378–409. 10.1111/j.1468-2885.2006.00275.x

[B3] Alves S. Martins A. Fonseca A. Canavarro M. C. Pereira M. (2018). Preventing and treating women’s postpartum depression: a qualitative systematic review on partner-inclusive interventions. *J. Child Fam. Stud.* 27 1–25. 10.1007/s10826-017-0889-z

[B4] American Psychiatric Association. (2013). Diagnostic and Statistical Manual of mental disorders (DSM-5). *Ind. J. Psychiatr.* 55 220–223. 10.4103/0019-5545.117131 PMC3777342 24082241

[B5] Badr H. Acitelli L. K. (2017). Re-thinking dyadic coping in the context of chronic illness. *Curr. Opin. Psychol.* 13 44–48. 10.1016/j.copsyc.2016.03.001 28813292

[B6] Baik O. M. Adams K. B. (2011). Improving the well-being of couples facing cancer: a review of couples-based psychosocial interventions. *J. Marital Fam. Ther.* 37 250–266. 10.1111/j.1752-0606.2010.00217.x 21457288

[B7] Berg C. A. Upchurch R. (2007). A developmental-contextual model of couples coping with chronic illness across the adult life span. *Psychol. Bull.* 133 920–954. 10.1037/0033-2909.133.6.920 17967089

[B8] Berg C. A. Wiebe D. J. Butner J. Bloor L. Bradstreet C. Upchurch R. (2008). Collaborative coping and daily mood in couples dealing with prostate cancer. *Psychol. Aging* 23 505–516. 10.1037/a0012687 18808241

[B9] Bergstraesser E. Inglin S. Hornung R. Landolt M. A. (2015). Dyadic coping of parents after the death of a child. *Death Stud.* 39 128–138. 10.1080/07481187.2014.920434 25204680

[B10] Bertschi I. C. Meier F. Bodenmann G. (2021). Disability as an interpersonal experience: a systematic review on dyadic challenges and dyadic coping when one partner has a chronic physical or sensory impairment. *Front. Psychol.* 12:624609. 10.3389/fpsyg.2021.624609 33732189 PMC7959177

[B11] Bodenmann G. (1995). A systemic-transactional conceptualization of stress and coping in couples. *Swiss J. Psychol.* 54 34–49. 10.1016/j.cpr.2008.10.004 19167139

[B12] Bodenmann G. (1997). Dyadic coping: a systemic-transactional view of stress and coping among couples: theory and empirical findings. *Eur. Rev. Appl. Psychol.* 47 137–140.

[B13] Bodenmann G. (2005). “Dyadic coping and its significance for marital functioning,” in *Couples coping with stress. Emerging perspectives on dyadic coping*, eds Revenson T. A. Kayser K. Bodenmann G. (Washington: American Psychological Association), 33–49. 10.1080/10615806.2021.1912740

[B14] Bodenmann G. (2008b). *Dyadisches Coping Inventar.* Bern: Huber.

[B15] Bodenmann G. (2008a). *Dyadic Coping Coding System. Unpublished Manual.* Switzerland: University of Zurich.

[B16] Bodenmann G. Charvoz L. Widmer K. Bradbury T. N. (2004). Differences in individual and dyadic coping among low and high depressed, partially remitted, and nondepressed persons. *J. Psychopathol. Behav. Assess.* 26 75–85.

[B17] Bodenmann G. Ledermann T. Bradbury T. N. (2007). Stress, sex, and satisfaction in marriage. *Personal Relationships* 14 551–569. 10.1111/j.1475-6811.2007.00171.x

[B18] Bodenmann G. Perrez M. (1991). Dyadisches Coping—Eine systemische Betrachtungsweise der Belastungsbewältigung in Partnerschaften [Dyadic coping—A systemic view of stress and coping in couples]. *Zeitschrift für Familienforschung* 3 4–25.

[B19] Bodenmann G. Pihet S. Kayser K. (2006). The relationship between dyadic coping and marital quality: a 2-year longitudinal study. *J. Fam. Psychol.* 20 485–493. 10.1037/0893-3200.20.3.485 16938007

[B20] Bodenmann G. Plancherel B. Beach S. R. H. Widmer K. Gabriel B. Meuwly N. (2008). Effects of coping-oriented couples therapy on depression: a randomized clinical trial. *J. Consul. Clin. Psychol.* 76 944–954. 10.1037/a0013467 19045963

[B21] Bodenmann G. Randall A. K. Falconier M. K. (2016). “Coping in couples: The Systemic Transactional Model,” in *Couples Coping with Stress: A Cross-Cultural Perspective*, eds Falconier M. K. Randall A. K. Bodenmann G. (Milton Park: Routledge), 5–22.

[B22] Buunk B. P. Berkhuysen M. A. Sanderman R. Nieuwland W. (1996). Actieve betrokkenheid, beschermend bufferen en overbescherming. *Gedrag & Gezondheid: Tijdschrift voor Psychologie en Gezondheid* 24 304–313.

[B23] Caspi A. Bolger N. Eckenrode J. (1987). Linking person and context in the daily stress process. *J. Personal. Soc. Psychol.* 52 184–195. 10.1037//0022-3514.52.1.184 3820071

[B24] Coyne J. C. Smith D. A. (1991). Couples coping with a myocardial infarction: a contextual perspective on wives’ distress. *J. Personal. Soc. Psychol.* 61 404–412. 10.1037/0022-3514.61.3.404 1941511

[B25] Cutrona C. E. (1996). *Social Support in Couples: Marriage as a Resource in Times of Stress.* Thousand Oaks: Sage.

[B26] Cutrona C. E. Bodenmann G. Randall A. K. Clavél F. D. Johnson M. (2018). “Stress, dyadic coping, and social support: Moving toward integration,” in *The Cambridge Handbook of Personal Relationships*, 2nd Edn, eds Vangelisti A. L. Perlman D. (Cambridge: Cambridge University Press), 341–352. 10.1017/9781316417867.027

[B27] Cutrona C. E. Suhr J. A. (1992). Controllability of stressful events and satisfaction with spouse support behaviors. *Commun. Res.* 19 154–174. 10.1177/009365092019002002

[B28] Dalstra J. Kunst A. Borrell C. Breeze E. Cambois E. Costa G. (2005). Socioeconomic differences in the prevalence of common chronic diseases: an overview of eight European countries. *Int. J. Epidemiol.* 34 316–326. 10.1093/ije/dyh386 15737978

[B29] DeLongis A. O’Brien T. (1990). “An interpersonal framework for stress and coping: An application to the families of Alzheimer’s patients,” in, eds Stephens M. A. P. Crowther J. H. Hobfoll S. E. Tennenbaum D. L. (London: Hemisphere Publishing Corp), 221–239.

[B30] Dohrenwend S. Dohrenwend B. P. (1974). *Stressful Life Events: Their Nature and Effects.* New York: Wiley.

[B31] Donato S. Parise M. Iafrate R. Bertoni A. Finkenauer C. Bodenmann G. (2014). Dyadic coping responses and partners’ perceptions for couple satisfaction: an actor-partner interdependence analysis. *J. Soc. Pers. Relat.* 32, 580–600. 10.1177/0265407514541071

[B32] Falconier M. K. Jackson J. B. Hilpert P. Bodenmann G. (2015a). Dyadic coping and relationship satisfaction: a meta-analysis. *Clin. Psychol. Rev.* 42 28–46. 10.1016/j.cpr.2015.07.002 26295276

[B33] Falconier M. K. Kuhn R. (2019). Dyadic coping in couples: a conceptual integration and a review of the empirical literature. *Front. Psychol.* 10:571–571. 10.3389/fpsyg.2019.00571 30971968 PMC6443825

[B34] Falconier M. K. Nussbeck F. Bodenmann G. (2013). Immigration stress and relationship satisfaction in Latino couples: the role of dyadic coping. *J. Soc. Clin. Psychol.* 32, 813–843. 10.1521/jscp.2013.32.8.813

[B35] Falconier M. K. Nussbeck F. Bodenmann G. Schneider H. Bradbury T. (2015b). Stress from daily hassles in couples: its effects on intradyadic stress, relationship satisfaction, and physical and psychological well-being. *J. Marital Fam. Ther.* 41 221–235. 10.1111/jmft.12073 24807831

[B36] Falconier M. K. Randall A. K. Bodenmann G. (eds) (2016). *Couples Coping with Stress: A Cross-Cultural Perspective.* Milton Park: Routledge.

[B37] Falconier M. K. Rusu P. P. Bodenmann G. (2019). Initial validation of the dyadic coping inventory for financial stress. *Stress and Health* 35 367–381. 10.1002/smi.2862 30821892

[B38] Ferlay J. Ervik M. Lam F. Colombet M. Mery L. Piñeros M. (2020). Global Cancer Observatory: Cancer Today. Geneva: WHO

[B39] Helgeson V. S. (1993). The onset of chronic illness: its effect on the patient-spouse relationship. *J. Soc. Clin. Psychol.* 12 406–428. 10.1521/jscp.1993.12.4.406

[B40] Helgeson V. S. Jakubiak B. Van Vleet M. Zajdel M. (2018). Communal coping and adjustment to chronic illness: theory update and evidence. *Personal. Soc. Psychol. Rev.* 22 170–195. 10.1177/1088868317735767 29053057 PMC5878976

[B41] Henrich J. Heine S. J. Norenzayan A. (2010). The weirdest people in the world? *Behav. Brain Sci.* 33 61–83. 10.1017/S0140525X0999152X 20550733

[B42] Hilpert P. Randall A. K. Sorokowski P. Atkins D. C. Sorokowska A. Ahmadi K. (2016). The associations of dyadic coping and relationship satisfaction vary between and within nations: a 35-nation study. *Front. Psychol.* 7:1106–1106. 27551269 10.3389/fpsyg.2016.01106 PMC4976670

[B43] Kayser K. Watson L. E. Andrade J. T. (2007). Cancer as a “we-disease”: examining the process of coping from a relational perspective. *Fam. Syst. Health* 25 404–418. 10.1037/1091-7527.25.4.404

[B44] Kuhn R. Bradbury T. N. Nussbeck F. W. Bodenmann G. (2018). The power of listening: lending an ear to the partner during dyadic coping conversations. *J. Fam. Psychol.* 32 762–772. 10.1037/fam0000421 29863374

[B45] Kuhn R. Milek A. Meuwly N. Bradbury T. N. Bodenmann G. (2017). Zooming in: a microanalysis of couples’ dyadic coping conversations after experimentally induced stress. *J. Fam. Psychol.* 31 1063–1073. 10.1037/fam0000354 29309189

[B46] Landis M. Peter-Wight M. Martin M. Bodenmann G. (2013). Dyadic coping and marital satisfaction of older spouses in long-term marriage. *GeroPsych* 26 39–47. 10.1024/1662-9647/a000077

[B47] Langer S. L. Rudd M. E. Syrjala K. L. (2007). Protective buffering and emotional desynchrony among spousal caregivers of cancer patients. *Health Psychol.* 26 635–643. 10.1037/0278-6133.26.5.635 17845115

[B48] Lau K. K. H. Randall A. K. Duran N. D. Tao C. (2019). Examining the effects of couples’ real-time stress and coping processes on interaction quality: language use as a mediator. *Front. Psychol.* 9:2598. 10.3389/fpsyg.2018.02598 30697175 PMC6340998

[B49] Lazarus R. S. Folkman S. (1984). *Stress, Appraisal, and Coping (11th ed.).* New York: Springer.

[B50] Leuchtmann L. Bodenmann G. (2017). Interpersonal view on physical illnesses and mental disorders. *Schweizer Archiv Für Neurologie, Psychiatrie Und Psychotherapie* 168 170–174.

[B51] Leuchtmann L. Zemp M. Milek A. Nussbeck F. W. Brandstätter V. Bodenmann G. (2018). Role of clarity of other’s feelings for dyadic coping. *Personal. Relationships* 25 38–49. 10.1111/pere.12226

[B52] Li Q. Loke A. Y. (2014). A systematic review of spousal couple-based intervention studies for couples coping with cancer: direction for the development of interventions. *Psycho-Oncology* 23 731–739. 10.1002/pon.3535 24723336

[B53] Liberati A. Altman D. G. Tetzlaff J. Mulrow C. Gøtzsche P. C. Ioannidis J. P. A. (2009). The PRISMA statement for reporting systematic reviews and meta-analyses of studies that evaluate health care interventions: explanation and elaboration. *Ann. Intern. Med.* 151 W65–W94. 19622512 10.7326/0003-4819-151-4-200908180-00136

[B54] Luo X. Gao L. Li J. Lin Y. Zhao J. Li Q. (2020). A critical literature review of dyadic web-based interventions to support cancer patients and their caregivers, and directions for future research. *Psycho-Oncology* 29 38–48. 10.1002/pon.5278 31702839

[B55] Lyons R. F. Mickelson K. D. Sullivan M. J. Coyne J. C. (1998). Coping as a communal process. *J. Soc. Personal. Relationships* 15 579–605. 10.1177/0265407598155001

[B56] Lyons R. F. Sullivan M. J. L. Ritvo P. G. Coyne J. C. (1995). *Relationships in Chronic Illness and Disability.* Thousand Oaks: Sage.

[B57] Martire L. M. Schulz R. Helgeson V. S. Small B. J. Saghafi E. M. (2010). Review and meta-analysis of couple-oriented interventions for chronic illness. *Ann. Behav. Med.* 40 325–342. 10.1007/s12160-010-9216-2 20697859 PMC4101802

[B58] Martos T. Szabó E. Koren R. Sallay V. (2019). Dyadic coping in personal projects of romantic partners: assessment and associations with relationship satisfaction. *Curr. Psychol.* 40 2956–2969. 10.1007/s12144-019-00222-z

[B59] Merz C. A. Meuwly N. Randall A. K. Bodenmann G. (2014). Engaging in dyadic coping: buffering the impact of everyday stress on prospective relationship satisfaction. *Fam. Sci.* 5, 30–37. 10.1080/19424620.2014.927385

[B60] Meuwly N. Davila J. (2021). Associations between internalized heterosexism and perceived and observed support in same-gender couples. *Couple Fam. Psychol. Res. Pract.* 11, 102–116. 10.1037/cfp0000199

[B61] Meuwly N. Feinstein B. A. Davila J. Nuñez D. G. Bodenmann G. (2013). Relationship quality among Swiss women in opposite-sex versus same-sex romantic relationships. *Swiss J. Psychol.* 72 229–233. 10.1024/1421-0185/a000115

[B62] Mitchell A. M. Owen J. Adelson J. L. France T. Inch L. J. Bergen C. (2015). The influence of dyadic coping in relationship education for low-income racial and ethnic minority couples. *J. Fam. Ther.* 37 492–508. 10.1111/1467-6427.12057

[B63] Neff L. A. Karney B. R. (2007). Stress crossover in newlywed marriage: a longitudinal and dyadic perspective. *J. Marriage Fam.* 69 594–607. 10.1111/j.1741-3737.2007.00394.x

[B64] Nguyen T. P. Karney B. R. Bradbury T. N. (2020). When poor communication does and does not matter: the moderating role of stress. *J. Fam. Psychol.* 34 676–686. 10.1037/fam0000643 32077736 PMC7438248

[B65] O’Brien T. B. DeLongis A. (1996). The interactional context of problem-, emotion-, and relationship-focused coping: the role of the big five personality factors. *J. Personal.* 64 775–813. 10.1111/j.1467-6494.1996.tb00944.x 8956513

[B66] Pagani A. F. Donato S. Parise M. Bertoni A. Iafrate R. Schoebi D. (2019). Explicit stress communication facilitates perceived responsiveness in dyadic coping. *Front. Psychol.* 10:401. 10.3389/fpsyg.2019.00401 30873090 PMC6400868

[B67] Peters M. D. J. Godfrey C. M. Khalil H. McInerney P. Parker D. Soares C. B. (2015). Guidance for conducting systematic scoping reviews. *Int. J. f Evid.-Based Healthcare* 13 141–146. 10.1097/XEB.0000000000000050 26134548

[B68] Randall A. K. Bodenmann G. (2009). The role of stress on close relationships and marital satisfaction. *Clin. Psychol. Rev.* 29 105–115. 10.1016/j.cpr.2008.10.004 19167139

[B69] Randall A. K. Leon G. Basili E. Martos T. Boiger M. Baldi M. (2022). Coping with global uncertainty: perceptions of COVID-19 psychological distress, relationship quality, and dyadic coping for romantic partners across 27 countries. *J. Soc. Personal Relationships* 39 3–33. 10.1177/02654075211034236

[B70] Randall A. K. Tao C. Totenhagen C. J. Walsh K. J. Cooper A. N. (2017a). Associations between sexual orientation discrimination and depression among same-sex couples: moderating effects of dyadic coping. *J. Couple Relationship Ther.* 16 325–345. 10.1080/15332691.2016.1253520

[B71] Randall A. K. Totenhagen C. J. Walsh K. J. Adams C. Tao C. (2017b). Coping with workplace minority stress: associations between dyadic coping and anxiety among women in same-sex relationships. *J. Lesb. Stud.* 21 70–87. 10.1080/10894160.2016.1142353 27611568

[B72] Regan T. W. Lambert S. D. Kelly B. Falconier M. K. Kissane D. Levesque J. V. (2015). Couples coping with cancer: exploration of theoretical frameworks from dyadic studies. *Psycho-Oncology* 24 1605–1617. 10.1002/pon.3854 26059915

[B73] Revenson T. A. (1994). Social support and marital coping with chronic illness. *Ann. Behav. Med.* 16 122–130.

[B74] Revenson T. A. DeLongis A. (2011). “Couples coping with chronic illness,” in *The Oxford Handbook of Stress, health, and Coping*, ed. Folkman S. (Oxford: Oxford University Press), 101–123.

[B75] Revenson T. A. Hagedoorn M. (2019). “Dyadic coping with illness,” in *The Cambridge Handbook of Psychology, Health and Medicine*, 3rd Edn, eds Llewellyn C. D. Ayers S. McManus C. Newman S. Petrie K. J. Revenson T. A. (Cambridge: Cambridge University Press), 118–122.

[B76] Rohrbaugh M. J. Mehl M. R. Shoham V. Reilly E. S. Ewy G. A. (2008). Prognostic significance of spouse we talk in couples coping with heart failure. *J. Consul. Clin. Psychol.* 76 781–789. 10.1037/a0013238 18837595

[B77] Sarno E. L. Bundy C. Dyar C. Newcomb M. E. (2021). Examining minority stress, dyadic coping, and internalizing symptoms among male same-sex couples using actor–partner interdependence models. *J. Counsel. Psychol.* 68 515–525. 10.1037/cou0000542 33749295 PMC8455724

[B78] Slatcher R. B. Pennebaker J. W. (2006). How do I love thee? Let me count the words: the social effects of expressive writing. *Psychol. Sci.* 17 660–664. 10.1111/j.1467-9280.2006.01762.x 16913946

[B79] Song C. Buysse A. Zhang W. Lu C. Zhao M. Dewaele A. (2020). Coping with minority stress in romantic relationships among lesbian, gay and bisexual people. *Curr. Psychol.* 10.1007/s12144-020-01188-z

[B80] Ştefǎnuţ A. M. Vintilǎ M. Tudorel O. I (2021). The relationship of dyadic coping with emotional functioning and quality of the relationship in couples facing cancer—A meta-analysis. *Front. Psychol.* 11:594015. 10.3389/fpsyg.2020.594015 33488460 PMC7819877

[B81] Story L. B. Bradbury T. N. (2004). Understanding marriage and stress: essential questions and challenges. *Clin. Psychol. Rev.* 23 1139–1162. 10.1016/j.cpr.2003.10.002 14729426

[B82] Tausczik Y. R. Pennebaker J. W. (2010). The psychological meaning of words: LIWC and computerized text analysis methods. *J. Lang. Soc. Psychol.* 29 24–54. 10.1177/0261927X09351676

[B83] Traa M. J. De Vries J. Bodenmann G. Den Oudsten B. L. (2015). Dyadic coping and relationship functioning in couples coping with cancer: A systematic review. *Brit. J. Health Psychol.* 20 85–114. 10.1111/bjhp.12094 24628822

[B84] Wang P. S. Aguilar-Gaxiola S. Alonso J. Angermeyer M. C. Borges G. Bromet E. J. (2007). Use of mental health services for anxiety, mood, and substance disorders in 17 countries in the WHO world mental health surveys. *Lancet* 370 841–850. 10.1016/S0140-6736(07)61414-7 17826169 PMC2847360

[B85] Weitkamp K. Feger F. Landolt S. A. Roth M. Bodenmann G. (2021). Dyadic coping in couples facing chronic physical illness: A systematic review. *Front. Psychol.* 12:722740. 10.3389/fpsyg.2021.722740 34759866 PMC8573212

[B86] Williamson H. C. (2020). Early effects of the COVID-19 pandemic on relationship satisfaction and attributions. *Psychol. Sci.* 31 1479–1487. 10.1177/0956797620972688 33151125 PMC7797601

[B87] World Health Organization. (2019). *International Statistical Classification of Diseases and Related Health Problems (11th ed.).* Geneva: WHO.

[B88] Xu W. Wang J. Schoebi D. (2019). The role of daily couple communication in the relationship between illness representation and fear of cancer recurrence in breast cancer survivors and their spouses. *Psycho-Oncology* 28 1301–1307. 10.1002/pon.5082 30946501

[B89] Zarski A.-C. Berking M. Fackiner C. Rosenau C. Ebert D. D. (2017). Internet-based guided self-help for vaginal penetration difficulties: Results of a randomized controlled pilot trial. *J. Sex. Med.* 14 238–254. 10.1016/j.jsxm.2016.12.232 28161080

